# Control of a chemical reaction (photodegradation of the p3ht polymer) with nonlocal dielectric environments

**DOI:** 10.1038/srep14620

**Published:** 2015-10-05

**Authors:** V. N. Peters, T. U. Tumkur, G. Zhu, M. A. Noginov

**Affiliations:** 1 Center for Materials Research, Norfolk State University, Norfolk, VA 23504.

## Abstract

Proximity to metallic surfaces, plasmonic structures, cavities and other inhomogeneous dielectric environments is known to control spontaneous emission, energy transfer, scattering, and many other phenomena of practical importance. The aim of the present study was to demonstrate that, in spirit of the Marcus theory, the rates of chemical reactions can, too, be influenced by nonlocal dielectric environments, such as metallic films and metal/dielectric bilayer or multilayer structures. We have experimentally shown that metallic, composite metal/dielectric substrates can, indeed, control ordering as well as photodegradation of thin poly-3-hexylthiophene (p3ht) films. In many particular experiments, p3ht films were separated from metal by a dielectric spacer, excluding conventional catalysis facilitated by metals and making modification of the nonlocal dielectric environment a plausible explanation for the observed phenomena. This first step toward understanding of a complex relationship between chemical reactions and nonlocal dielectric environments is to be followed by the theory development and a broader scope of thorough experimental studies.

## Introduction

### Control of physical phenomena with inhomogeneous dielectric environments

#### 

It is well known that the intensity, the rate, the directionality, and the spectra of spontaneous emission can be controlled by local and nonlocal inhomogeneous dielectric environments, including but not limited to vicinity to plasmonic nanostructures^1^, metamaterials^2^,^3^, metallic surfaces^2^, and cavities^4^. These phenomena are often described in terms of enhanced density of photonic states^2^, which has also been shown to control the reflection and scattering^5^. However, the effect of inhomogeneous dielectric environments on a plethora of physical phenomena reaches far beyond the density of photonic states. Thus, in a known textbook example, interaction of two static point charges is modified by the presence of a mirror. We have recently demonstrated that the rate of the Förster energy transfer – the process dominated by the dipole-dipole interaction, whose rate strongly decreases with an increase of dielectric permittivity – is strongly inhibited in vicinity of metallic surfaces and lamellar metal/dielectric metamaterials^6^. In this work we take one step further and show that vicinity to metallic and metamaterial surfaces can control chemical reactions as well. The heuristic reasoning for this inference is outlined below.

### Marcus theory: Effect of dielectric environment on the rate of a charge transfer reaction

#### 

**Figure 1:**
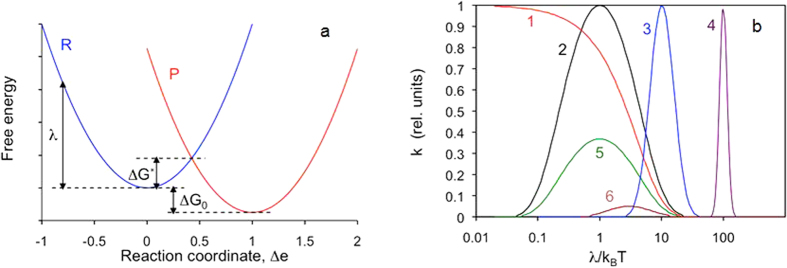
(**a**) Free energy of reactants plus environment (R curve) and free energy of products plus environment (P curve) plotted vs. reaction coordinate Δe. (Adopted and modified from ref. 9). (**b**) The exponent in Eq. 2 (∝*k*) plotted as a function of *λ*/*k*_*B*_*T* for Δ*G*_*0*_/*k*_*B*_*T* = 0 (1), Δ*G*_*0*_/*k*_*B*_*T* = −1 (2), Δ*G*_*0*_/*k*_*B*_*T* = −10 (3), Δ*G*_*0*_/*k*_*B*_*T* = −100 (5), Δ*G*_*0*_/*k*_*B*_*T* = 1 (5), and Δ*G*_*0*_/*k*_*B*_*T* = 3 (6).

The effect of a homogeneous dielectric environment on chemical reactions can be explained in an example of the Nobel Prize winning Marcus theory designed to describe redox reactions in a solution^7^,^8^,^9^,^10^. In simple terms, two reactants, donor and acceptor, have certain spatial distribution of electrical charges, which is balanced by an appropriate polarization of surrounding solvent molecules. Transfer of an electron from donor to acceptor changes the local charge distribution and causes re-polarization of solvent molecules. This change of polarization requires a so-called reorganization energy *λ*. It contributes to the potential barrier ΔG^*^, which the reaction should overcome as it moves along the reaction path (proportional to the amount of transferred charge Δ*e*) from the valley representing donor and acceptor before the reaction (reactants) to the valley representing the same agents after the reaction (products), Fig. 1a^9^.

Electric field in a dielectric is inversely proportional to the dielectric permittivity *ε*. Therefore, the reorganization energy is expected to decrease with increase of *ε*. Furthermore, polarization of solvent molecules has a fast electronic component (characteristic of visible or ultraviolet light frequency) and a slow ionic reorientation component (characterized by a microwave or radio frequency). Correspondingly, the dielectric permittivities at both characteristic frequencies enter the equation for the reorganization energy^9^,^10^,
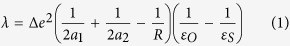
where *a*_*1*_ and *a*_*2*_ are radii of the reacting molecules (modeled as spheres), *R* is the distance between the centers of the molecules, *ε*_*o*_ is the dielectric permittivity at optical frequency, and *ε*_*s*_ is that at low frequency (nearly static).

The reorganization energy enters the formula for the reaction rate constant *k* as^9^,^10^,
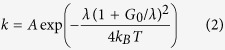
where A is the pre-exponential factor depending on the nature of the electron transfer reaction, *k*_*B*_ is the Boltzmann constant, *T* is the temperature, and Δ*G*_*0*_ is the standard free energy of the reaction. Here, for simplicity (following ref. 9), we have omitted vibrational component of the reorganization energy as well as the work terms, which are involved in bringing the reactants together and separating the reaction products.

In Fig. 1b, the reaction rate constant *k* is plotted as a function of *λ/k*_*B*_*T* for several different ratios Δ*G/k*_*B*_*T*. At Δ*G*_*0*_ = −*λ*, the exponent in Eq. 2 is equal to 1, and the reaction is activationless. At |Δ*G*_*0*_| > *λ* and |Δ*G*_*0*_| < *λ*, the reaction constant *k* strongly depends on *λ*, in particular, at |Δ*G*_*0*_|>>*k*_*B*_*T*, Fig. 1b. Depending on the value of the ratio |Δ*G*_*0*_|/*λ*, an increase of *λ* can lead to both increase or decrease of *k*. Under certain set of assumptions (which are not universal), the pre-exponential factor *A* is proportional to *λ*^−1/2^ 10. However, the behavior of the reaction rate constant *k* is still dominated by a much stronger exponential function and the curves in Fig. 1b remain almost unaltered.

The Marcus model, briefly outlined above, was originally developed for redox reactions in homogeneous dielectric media (solvents) and later extended to photosynthesis^10^, corrosion^11^, chemiluminescence^12^, and charge separation in organic solar cells^13^. In this work we infer that the reorganization energy and the rates of chemical reactions can also be controlled by non-local modifications of inhomogeneous dielectric environments. This motivated our study focused on experimental verification of this heuristic hypothesis.

### Chemical reaction: photo-oxidation of the p3ht polymer

#### 

**Figure 2:**
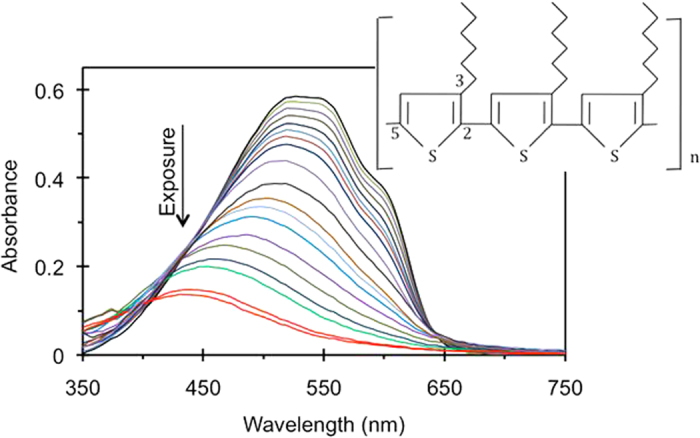
Absorbance spectra (natural logarithm) of the 23 nm thick regioregular p3ht film deposited onto the glass substrate with thick Ag film on the back (measured in the reflection experiment). The shoulder at ~610 nm and two (not well resolved) peaks at ~525 nm and ~555 nm are the signature of a quasi-ordered structure. Inset: Regioregular 2,5-poly-3-hexyl-thiophene (p3ht) polymer.

The chemical reaction in our study involved the semiconducting polymer, poly-3-hexylthiophene (p3ht), which is the material of choice in organic photovoltaics^14^. Specifically, 2,5-poly-3-hexyl-thiophene used in our study consists of hexylsubstituted (in position 3) thiophene monomers connected in positions 2 and 5 and forming a backbone of the polymer, inset of Fig. 2. The absorption spectrum of the regioregular p3ht is dominated by the band with the maximum at ~0.55 μm, which is characteristic of a chain of thiophene rings^15^,^16^. The polymeric chains tend to arrange in lamellar layers, and the polymer has a quasi-ordered structure^17^, which manifests itself in several peaks and shoulders seen on top of the p3ht absorption band, Fig. 2. This effect is particularly strong in annealed samples^18^.

Unfortunately, p3th is known for its strong photo-degradation in presence of oxygen, which makes it less attractive for device applications. Two mechanisms of photo-oxidation have been identified in the literature: (i) by a radical chain and (ii) by singlet oxygen^19^,^20^. In the former process, oxygen-centered radicals are believed to attack the α-carbon atom of the alkyl side chain by hydrogen abstraction, causing chain scission and photobleaching^19^,^21^. The corresponding action spectrum (spectral sensitivity of photodegradation) rises toward the ultraviolet (UV) part of the spectrum. In the second mechanism, singlet oxygen (whose formation is sensitized by photoexcitation of p3ht) attacks the π-electron system of the polymer backbone, which leads to photobleaching without affecting the side chains^19^,^22^. In this case, the action spectrum of photo-oxidation follows the absorption spectrum of the polymer, because it acts as a sensitizer for the oxidizing agent.

As both photo-oxidation mechanisms destroy thiophene rings and lead to reduction of the polymer absorption band, shortening of the polymer chains causes blue shift of the absorption band, which becomes particularly pronounced when the number of monomer units forming the chain is getting smaller than 10 (Ref. 18). At photoexposure with visible light corresponding to the maximum of the p3ht absorption (525 nm, singlet oxygen mechanism), the same amount of photodegradation (reduction of absorption) corresponds to a relatively large frequency shift as compared to that at UV photoexposure (365 nm, radical mechanism)^19^,^23^. This indicates that the former mechanism generates larger number of short p3ht chains, which absorb at shorter wavelengths. The difference in concentration of short polymeric chains produced by two photodegradation processes is, reportedly, because of the two reasons (i) the singlet oxygen mechanism favors random scission more than the radical mechanism does and (ii) visible light is not efficiently absorbed by short chains and does not destroy them^23^. Note that the magnitude of the frequency shift can be used to differentiate between the radical and the singlet oxygen mechanisms of the photodegradation.

## Experimental Samples and Measurements

### Experimental samples

**Figure 3:**
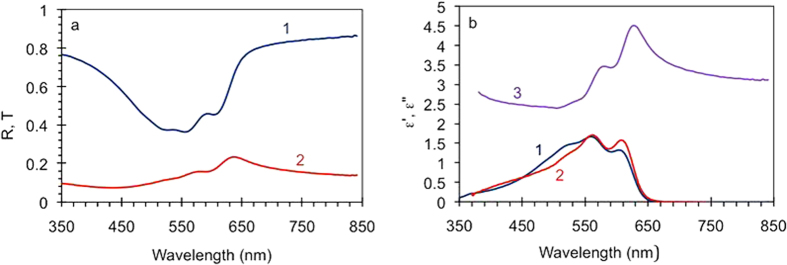
(**a**) Transmission (1) and reflection (2) spectra of the 78 nm thick p3ht film. (**b**) Spectra of imaginary *ε”* (trace 1) and real *ε’* (trace 3) parts of dielectric permittivity of p3ht determined from the transmission and reflection spectra. Trace 2 - spectrum of *ε”* calculated from trace 3 using the Kramers-Kronig relations.

Our experimental samples were thin films of p3ht (13 nm to 41 nm) deposited on a variety of substrates (see Methods). The spectra of real *ε’* and imaginary *ε”* parts of dielectric permittivity of p3ht were determined in the transmission and reflection experiments, Fig. 3a (see Methods). The determined spectra of *ε’* and *ε”* matched each other reasonably well *via* the Kramers Kronig relations, which made us confident in their accuracy, Fig. 3b.

The substrates included (1) glass slides, some of which had Ag mirror deposited on their back side; (2) MgF_2_ films deposited on glass, 35 nm and 75 nm; (3) silver, gold, and aluminum films, 50 nm to 280 nm; (4) nominally the same (as above) Ag, Au, and Al films with ~35 nm MgF_2_ film deposited on top; and (5) Ag/MgF_2_ lamellar metamaterials with Ag layer on top and with MgF_2_ layer on top (see Methods). The thicknesses of the layers in the Ag/MgF_2_ metamaterial were 25 nm for metal and 35 nm for the dielectric. When the metamaterial is described in the effective medium approximation^24^, its real part of dielectric permittivity in the direction parallel to the layers *ε*_||_ changes sign from positive to negative at *λ* ≈ 370 nm^25^. Above this critical wavelength, the metamaterial has hyperbolic dispersion (for extraordinary waves) and broad-band singularity of the density of photonic states^26^,^27^.

### Photoexposure and photodegradation

In most of our experiments, the samples were exposed to a white light illumination by a 50 W halogen lamp. The distance between the lamp and the sample was ~14 cm. The total exposure time was ≥100 hours. During this period, the absorbance spectra of the p3th films were measured multiple times in the transmission and reflection experiments (see Methods). After proper normalization to the reflection of the substrate, the latter spectra were equivalent to transmission spectra of the p3ht films of double thickness. The exposure intervals between the measurements ranged from one hour to 10 hours.

Sample fabrication, characterization, photo-exposure, and spectra acquisition experiments have been repeated several times, and p3ht films deposited on most types of the substrates have been studied in three independent sets of measurements. A representative series of the p3ht absorbance spectra corresponding to different illumination exposures, similar to those reported in the literature^18^,^19^,^20^,^21^,^22^,^23^, is shown in Fig. 2.

**Figure 4:**
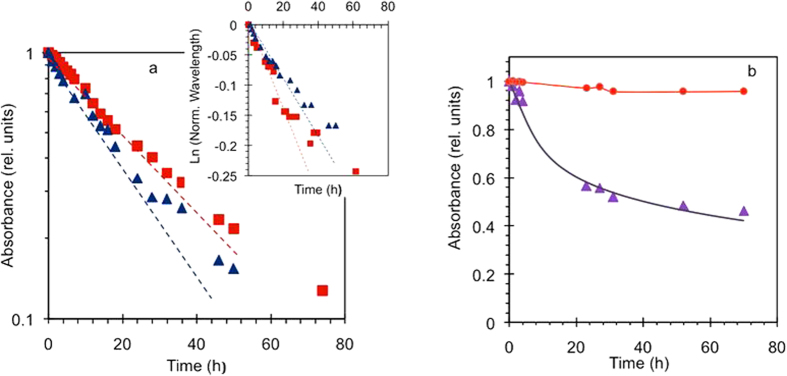
(**a**) Dependence of the maximal absorbance on the exposure time in the p3ht films deposited on glass (squares) and Ag/MgF_2_ metamaterial with Ag on top (triangles). Inset: Dependence of the (normalized to one) wavelength of the absorption maximum on the exposure time, measured in the same samples as in the main frame of Fig. a. (**b**) Time dependence of the maximal absorbance of the photoexposed p3ht (triangles) and the control sample kept in dark (circles). All data sets are normalized by the corresponding initial value before the photoexposure. Solid lines – guides for eye.

In agreement with numerous reports^18^,^19^,^20^,^21^,^22^,^23^, with increase of the light exposure, the absorption band is getting smaller (due to reduced number of survived thiophene rings) and its maximum position experiences a blue shift (due to shortening of the π conjugated p3ht polymer chains). When the absorbance and the wavelength (or the frequency) corresponding to the maximum of the absorption band were plotted against the exposure time, they resulted, respectively, in the absorption decay kinetics (Fig. 4a) or the spectral shift kinetics (inset of Fig. 4a).

At relatively short exposure times (≤30 hours) and drop of the p3ht peak absorption by approximately the factor of e, the absorption decay kinetics looked nearly linear in semi-logarithmic coordinates (linear time and logarithmic absorbance), suggesting quasi-exponential character of the process. The slope of this initial part of the kinetics was used to determine the absorption decay rate *W*_*a*_, which is proportional to the rate of destruction of thiophene rings^19^. (The decay slightly slowed down at longer exposure times, ≥40 hours). The rates of the spectral shift *W*_*s*_ have been determined in a similar way.

In the control measurement, we have compared degradation of the photoexposed sample with that of the sample kept in dark (Fig. 4b) and found the latter to be almost negligible, also in a good agreement with the literature^18^.

### Spectrally selective measurements and action spectrum of photodegradation

**Figure 5:**
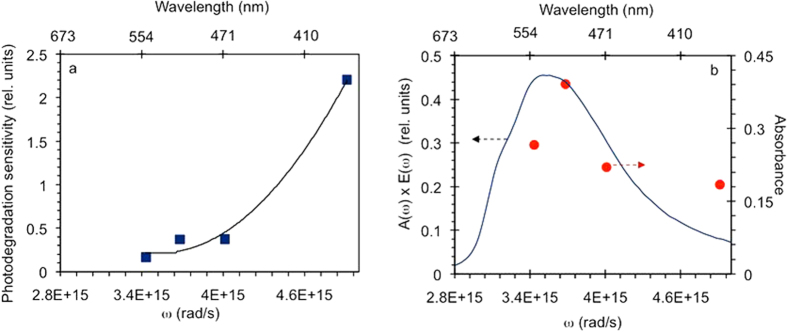
(**a**) Photodegradation sensitivity (action spectrum of photodegradation) *A*(*ω*) *–* squares; solid line – guide for eye. (**b**) Action spectrum *A*(*ω*) multiplied by the spectral emissivity of the halogen lamp *Ξ*(*ω*) (circles); solid line – absorbance spectrum of p3ht.

The following particular experiment was designed to determine the contributions of different spectral ranges to the overall photodegradation of p3ht. In this study, four nominally identical p3ht films deposited on glass were illuminated through band-pass color filters, whose transmittance maxima were equal to 350 nm, 430 nm, 485 nm, and 540 nm (see Methods). The corresponding absorbance decay rates *W*_*a*_(ω), measured as discussed above and properly normalized to the lamp emissivity and filter transmission, resulted in the action spectrum of the photodegradation *A*(*ω*) (Fig. 5a), which showed strong increase toward UV wavelengths, in agreement with refs 18,23. However, the same action spectrum multiplied by the lamp emissivity *Ξ*(*ω*) approximately followed the absorbance spectrum of p3ht at *λ* ≥ 450 nm and rose only slightly toward UV wavelengths, Fig. 5b. This suggests that in our experiments, we had contributions from both singlet oxygen and radical photodegradation mechanisms (as explained in Introduction).

### Correction for interference effects, spectral sensitivity of photodegradation, and lamp emissivity

**Figure 6:**
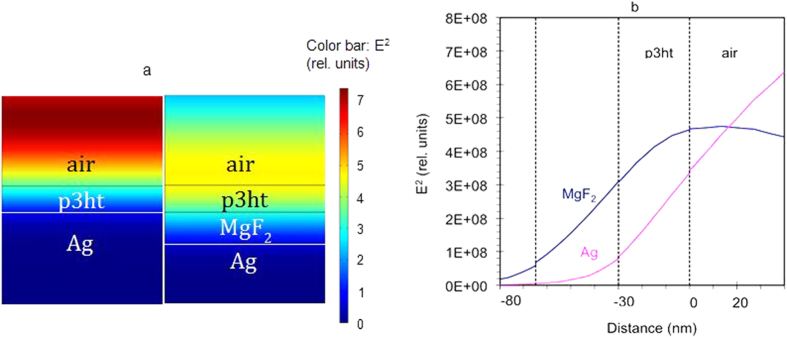
(**a**) Spatial distribution of the electric field squared, 
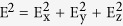
 [V^2^/cm^2^], calculated for the Ag/MgF_2_/p3ht/air structure (right panel) and the Ag/p3ht/air structure (left panel). (**b**) Electric field squared, E^2^, plotted as the function of the position for the Ag/MgF_2_/p3ht/air and Ag/p3ht/air structures. All calculations are done for normal incidence. One can see that the light intensity inside the p3ht film strongly depends on the presence of the MgF_2_ spacer.

Most of p3ht samples in our experiments were deposited onto reflecting substrates. The reflected light interfered with the incident light, producing a standing wave characterized by a series of bright and dark fringes above the sample’s surface, Fig. 6a. The positions of the fringes and, more generally, the distribution of electric field intensity as the function of the distance from the sample’s surface depended on the wavelength, the nature of the substrate (phase shift at reflection), as well as the thickness and dielectric permittivity of the p3ht film. If electric field intensity in the volume of the p3ht film was large (the film was positioned in the bright fringe of the interference pattern), the power of electromagnetic radiation absorbed by the polymer was large too (right panel of Fig. 6a and trace 1 in Fig. 6b). This resulted in large p3ht absorbance measured in the reflectance experiment and fast photodegradation. Correspondingly, small electric field intensity in the p3ht film resulted in small absorbed power, small measured absorbance, and slow photodegradation (left panel of Fig. 6a and trace 2 in Fig. 6b).

To recap, the rate of photodegradation depended not only on the lamp intensity (which was the same for all photoexposed samples), but also on the details of constructive and destructive interference and positions of the bright and dark fringes. Correspondingly, the measured absorbance of the p3ht films was determined not only by the material’s properties and the film thickness, but also by the positions of bright and dark fringes in a particular reflection experiment – the effect, which is only possible in samples of subwavelength thickness (<<*λ*).

Using COMSOL Multiphysics, we have calculated the reflection spectra and corresponding distributions of electric field intensity for the p3ht films of different thickness deposited on a variety of substrates used in our experiments. The comparison of the calculated and the experimentally determined values of absorbance (at *λ* = 550 nm) provided an independent method of evaluating thicknesses of the p3ht films.

We further normalized the experimentally determined rates of photodegradation *W*_*a*_ and spectral shift *W*_*s*_ to (i) the light intensity inside p3ht films (originating from interference of incident and reflected waves) and (ii) the sensitivity of photodegradation to different frequencies in the emissivity spectra of our halogen lamp (see Methods). All rate constants described below are normalized for the standing wave interference effect, spectral sensitivity of photodegradation, and lamp emissivity.

## Results

### Effect of substrate on the crystallinity of p3ht

It has been reported in the literature^16^,^18^ that annealed p3ht films have high crystallinity, which manifests itself in two pronounced maxima at 554 nm 525 nm as well as the shoulder at 610 nm. At the same time, not annealed samples have, as a rule, lower crystallinity, which results in much smoother absorption spectra with less pronounced shoulders and the absorption maximum at ~520 nm^18^.

**Figure 7:**
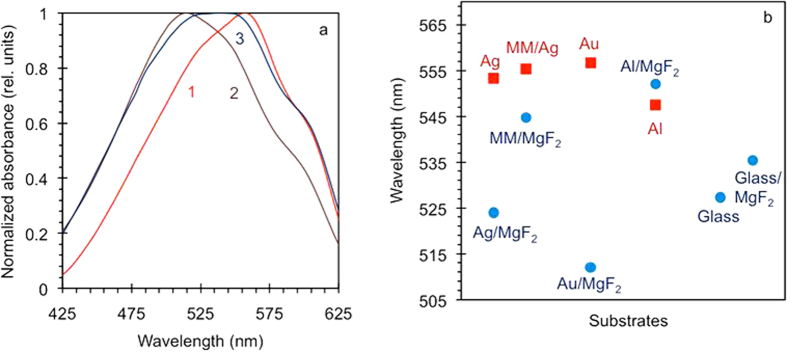
(**a**) Absorption spectra of not photoexposed p3ht films deposited on Ag/MgF_2_ metamaterial with Ag on top (1), Ag film with MgF_2_ on top (2), and Ag/MgF_2_ metamaterial with MgF_2_ on top. (**b**) Maxima of the p3ht absorption bands in samples deposited onto a variety of substrates.

In our not annealed p3ht films (before the photoexposure), we have observed the absorbance spectra with the maxima at ~555 nm (e.g. trace 1 in Fig. 4a) as well as at much shorter wavelengths (traces 2 and 3 in Fig. 7a). In Fig. 7b, we have summarized the wavelengths of the absorption maxima in the p3ht films deposited on Ag, Au, Al, and lamellar Ag/MgF_2_ metamaterial (with Ag as the outmost layer) and the wavelengths of the absorption maxima on the same substrates with ~35 nm MgF_2_ layer on top. The wavelengths of the p3ht absorption maxima on MgF_2_ and glass are shown in the figure as well. (The data were averaged over several nominally identical samples when appropriate.) One can see that almost all data points corresponding to the substrates with metal on top are higher than those in the samples with dielectric on top, Fig. 7b. This brings us to the first experimental observation of this study.

#### Observation 1

The p3ht films in direct contact with metal have higher wavelength positions of their absorption maxima than the films, which are deposited on purely dielectric substrates or separated from metal by a layer of MgF_2_. The only exception from this rule are the data points corresponding to Al based samples, in which the effect of the dielectric spacer on the wavelength position of the absorption maximum was very small. This finding suggests that direct contact with Ag and Au affects crystallinity ordering of the p3ht polymer. Whether this phenomenon is directly related to an inhomogeneous dielectric environment, as discussed in Introduction, remains an open question.

Reportedly, the degree of crystallinity of p3ht does not affect the rate of its photodegradation^18^ and should not affect the results described below.

### Rates of the reduction of absorption and of the frequency shift

**Figure 8:**
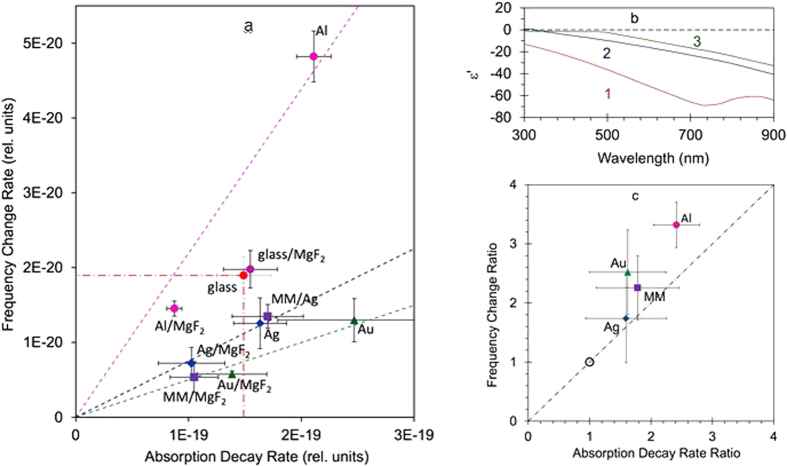
(**a**) The rate of the reduction of absorbance plotted against the rate of the frequency shift of the absorption maximum. The rates are normalized to the corresponding effective pumping intensities (coefficients *K* in Methods). (**b**) Spectra of real parts of dielectric permittivities in Al^34^ (trace 1), Ag^33^ (trace 2), and Au^33^ (trace 3). (**c**) Vertical axis: absorption decay rates measured on substrates with metal on top normalized by those measured on their counterparts with MgF_2_ on top. Horizontal axis: same for frequency shift rates. The (1, 1) point represents all substrates with MgF_2_ on top.

For all samples studied, we plotted the rate of the reduction of absorbance *versus* the rate of the frequency shift of the absorption maximum, Fig. 8a. All rates were normalized to the corresponding effective pumping intensities, lamp emissivity, and spectral sensitivity of photodegradation (coefficients *K* in Methods). When appropriate, the data points were averaged over several measurements done in nominally equivalent samples. Error bars are indicated for all data points. This figure allows us to make several important observations.

#### Observation 2

Photodegradation rates of p3ht films deposited on glass and on MgF_2_ films on top of glass were nearly the same (the “glass/MgF_2_” point in Fig. 8a is averaged over 75 nm and 35 nm MgF_2_ films deposited on glass). Therefore, neither chemical nature of the dielectric substrate nor its index of refraction (1.38 for MgF_2_ and 1.50 for glass) played any significant role in photodegradation.

#### Observation 3

In first approximation, a linear trendline (crossing the origin) can be drawn through each couple of data points corresponding to the same type of substrate with and without MgF_2_ layer on top (e.g. Ag and Ag/MgF_2_, etc.). One can see that these slopes are substantially different for Au based substrates, Ag based substrates, and Al based substrates. High slope of the trendline in Al based samples manifests a relatively fast frequency shift at relatively slow reduction of absoroption, which is characteristic of a singlet oxygen photodegradation mechanism triggered by visible light directly absorbed by p3ht rings (see Introduction). At the same time, small value of the slope in Au based samples corresponds to a relatively slow frequency shift at relatively fast reduction of absorption, which is characteristic of a radical photodegradation mechanism triggered by a UV light. The slope for the Ag based samples (which is very close to that for Ag based metamaterials) is in between for those in Au and Al. It is interesting to note that the ordering of the slopes in the Au, Ag, and Al based samples corresponds to the ordering of the spectra of real parts of dielectric permittivity, Fig. 8b.

#### Observation 4

All data points corresponding to metal-based substrates with MgF_2_ on top are closer to the origin than the data points measured on glass and MgF_2_ without metal (see the points inside the dash-dotted box, whose outer corner corresponds to the data point on glass, Fig. 8a).

#### Observation 5

All data points corresponding to metal-based substrates with MgF_2_ on top are closer to the origin than their counterparts measured on the same substrates without MgF_2_ spacer. This effect can be partly due to a conventional catalysis facilitated by metals^28^,^29^,^30^.

#### Observation 6

The linear trendlines drawn in Fig. 8a through pairs of points corresponding to the same substrates with and without MgF_2_ on top, were only a rough fit to the experimental data. This can be seen in Fig. 8c, in which both the absorption decay rate and the frequency shift rate measured on substrates with metal on top were divided by those measured in their counterparts with MgF_2_ on top. The (1, 1) point on the graph represents all substrates with MgF_2_ on top. The fact that all points corresponding to samples with metal on top lie above the y = x line, leads us to the conclusion that although direct contact with metal accelerates both the reduction of absorption and the spectral shift, its effect on the frequency shift is larger than on the reduction of absorption. This suggests that the contact with metal has a stronger effect on the singlet oxygen degradation mechanism than on the radical degradation mechanism.

## Discussion and Summary

To summarize, we have studied photodegadation of the semiconducting p3ht polymer deposited on a variety of dielectric, metallic and metal/dielectric substrates. We found that: (1) Direct contact with metal affects absorption spectra of p3ht films, which indicates change of their crystallinity. (2) Purely dielectric substrate (glass or MgF_2_) has no noticeable effect on photodegadation of p3ht. (3) The nature of metal-based substrate affects the ratio between the rate of the absorption reduction and the rate of the frequency shift. This can be interpreted as changing the ratio between the singlet oxygen and the radical mechanisms of photodegradation. (4) The rates of photodegradation (reduction of absorption and frequency shift) in p3ht films deposited on metallic substrates with MgF_2_ on top are smaller than in those deposited onto purely dielectric substrates. We infer that this result manifests experimental evidence of inhibition of a chemical reaction by a nonlocal dielectric environment. (5) The rates of photodegradation in p3ht films deposited directly onto the metallic substrates are higher than in those in the corresponding samples with the MgF_2_ spacer separating metal and p3ht. (6) Direct contact with metal increases the rate of the frequency shift stronger than it does increase the rate of the absorption reduction (preferential enhancement of the singlet oxygen degradation mechanism). Note that the vicinity to metallic surfaces and lamellar metal-dielectric metamaterials can potentially modify the p3ht excited state decay time and, correspondingly, affect the rate of the photodegradation. The study of this mechanism is in progress and its results will be published elsewhere.

Some of the experimental observations above, *e.g.* #1 and #5, can be partly due to a conventional catalysis. However, the results related to p3ht films, which were not in direct contact with metal, call for an explanation that extends beyond the catalysis. As it has been discussed in Introduction, inhomogeneous and nonlocal dielectric environments are expected to affect the rates of chemical reactions. Therefore, a vicinity to metallic layers can explain some of our findings. The photodegradation rates discussed in this work (e.g. those in Fig. 8a) are related to the reaction rate constant *k*. What remains unknown for p3ht is (i) the relationship between the nonlocal dielectric environment and effective reorganization energy *λ* and (ii) the relationship between *λ* and *k*. Co-existence of two photodegradation mechanisms complicates the analysis even further. We hope that the reported experimental results will stimulate new theoretical efforts.

Retrospectively, in studies of nonlocal dielectric environments on chemical reactions, a better understanding of the relationship between the theory and the experiment can be sought if the chosen chemical reactions are very simple, not photoinduced, and their mechanisms are very well known.

## Methods

### Experimental samples

Our experimental samples were thin films of p3ht deposited on a variety of substrates. The polymer (98% head to tail regioregular, average M_n_ = 54,000–75,000, purchased from Sigma Aldrich) was dissolved in chloroform, 3 mg/ml. The solution was spin coated onto the substrates and dried in air for 2 hours (avoiding any light exposure). Any high temperature annealing had to be avoided in order to prevent silver-based substrates from oxidation and damage. The film thickness (ranging from 13 nm to 41 nm) has been determined in direct profilometer measurements (Dektak XT, Bruker) as well as from the transmission and reflection measurements, as discussed in the Experimental Samples and Measurements section.

The substrates for p3ht films included metallic films, MgF_2_ films, and lamellar metal-dielectric multilayered structures (see Experimental Samples and Measurements). All layers were deposited using thermal vapor deposition technique. The film thickness was measured with the profilometer.

### Dielectric permittivity of p3ht

Experimentally, the 78 nm p3ht film has been deposited on glass and its reflection and transmission spectra have been measured in the spectrophotometer equipped with an integrated sphere (Lambda 900 from Perkin Elmer), Fig. 3a. The spectra of real *ε’* and imaginary *ε”* parts of dielectric permittivity have been determined by numerically solving an inverse problem^31^, using the known formulas relating the transmittance *T* and reflectance *R* to *ε’* and *ε”*^32^, Fig. 3b. (Note that the pairs of values *ε’* and *ε”* have been determined for each spectral point without relying on any model, such as Drude or Lorentz, relating to each other dielectric permittivities at different wavelengths).

### Photodegradation measurements

In the photodegradation experiments, using the same spectrophotometer setup as described above, we have measured transmission spectra of the p3ht films deposited onto transparent substrates and reflection spectra of the samples deposited onto opaque mirror-like substrates. After proper normalization to the reflection of the substrate (which was typically measured in almost completely photobleached samples after ≥100 hour exposure), the latter spectra were equivalent to transmission spectra of the p3ht films of double thickness. The experiments were carried out in an ambient room environment at typical temperature and humidity equal to ~23 °C and~35% respectively.

### Spectrally resolved photodegradation measurements

In this particular experiment, four nominally identical ~20 nm p3ht films deposited on glass were photoexposed through band-pass color filters, whose transmittance maxima were equal to 350 nm, 430 nm, 485 nm, and 540 nm. The corresponding absorbance decay kinetics, acquired as discussed above and in the Experimental Samples and Measurements section, were used to determine the absorbance decay rates *W*_*a*_(ω), which were proportional to the action spectrum of the photodegradation *A*(*ω*), the lamp emissivity *Ξ*(*ω*), and the integrated transmission spectra of the color filter 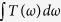, *W*_*a*_(ω) = *A*(*ω*)*Ξ*(*ω*) 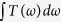. The emissivity spectrum of the halogen lamp *Ξ*(*ω*) was measured with the help of the calibrated lamp (model 68831, Oriel/Newport), monochromator (model MS257, Oriel/Newport), and a photomultiplier (model R5108, Hamamatsu). This allowed us to derive the action spectrum of the photodegradation *A*(*ω*) (Fig. 5a) as well as the action spectrum multiplied by the lamp emissivity *Ξ*(*ω*) (Fig. 5b), as discussed in the Experimental Samples and Measurements section.

### Normalizations

Using COMSOL Multiphysics software package, we have calculated the reflection spectra and corresponding distributions of electric field intensity for the p3ht films of different thickness deposited on a variety of substrates. In calculations we used the spectra of dielectric permittivity of p3ht depicted in Fig. 3a, the spectra of dielectric permittivity of Ag and Au were taken from ref. 33, and those of Al and MgF_2_ were found in refs 34,35, respectively.

Our next task was to normalize the experimentally determined rates of photodegradation *W*_*a*_ and spectral shift *W*_*s*_ to (i) the light intensity inside p3ht films (originating from interference of incident and reflected waves) and (ii) the sensitivity of photodegradation to different frequencies in the emissivity spectra of our halogen lamp. In order to accomplish this goal, we calculated the electric field squared 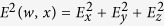, which is proportional to the light intensity *I*(*ω, x*)) for different frequencies *ω* and different locations inside the film *x* (measured from the polymer/air interface) for the thicknesses of the p3ht films *d* corresponding to those in our experiments, Fig. 6b.

We then integrated the intensities over the film thickness 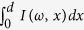 and multiplied this spectrum by the action spectrum *A*(ω) and the emissivity spectrum of the halogen lamp *Ξ*(ω). The resultant function 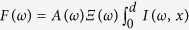 *dx* took into account the interference effects, the spectral sensitivity of photodegradation (action spectrum), and the spectrum emissivity of our lamp. By integrating this function between ω_1_ = 2.9 × 10^15^ rad/s (*λ*_1_ = 650) nm, which approximately corresponded to the long wavelength limit of the p3ht absorption spectrum, and ω_2_ = 5.8 × 10^15^ rad/s (*λ*_2_ = 325 nm), which approximately corresponded to onset of the lamp’s emission in the UV part of the spectrum, we have obtained the integrated normalization constant 
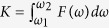
. This constant, which was different for different substrates and thicknesses of the p3ht film, was further used to normalize the experimentally measured rates of the absorption decay and the spectral shift.

## Additional Information

**How to cite this article**: Peters, V. N. *et al.* Control of a chemical reaction (photodegradation of the p3ht polymer) with nonlocal dielectric environments. *Sci. Rep.* **5**, 14620; doi: 10.1038/srep14620 (2015).
